# A Review of Synchronous Findings of Hypothyroidism and Cholelithiasis

**DOI:** 10.7759/cureus.30316

**Published:** 2022-10-14

**Authors:** Varun Kulkarni, Harshal Ramteke, Yashwant Lamture, Pankaj Gharde

**Affiliations:** 1 Department of General Surgery, Jawaharlal Nehru Medical College, Datta Meghe Institute of Medical Sciences, Wardha, IND

**Keywords:** bile, sphincter of oddi, cholecystectomy, cholelithiasis, hypothyroidism

## Abstract

Cholelithiasis is considered to be the most common biliary pathology. They have been categorized into three types, which are pigment stones, cholesterol stones, and mixed types of stones with varying incidence. The condition may be asymptomatic for significantly long durations and in most cases, the presence of gall stones is an incidental finding. The patients may present with pain in the abdomen in stages of cholecystitis or advanced stages or cases of gall stones causing the obstruction. Gallbladder stones are formed through a very complex procedure with the contribution of numerous factors, where the main initiating step is supposed to be the development of a state wherein there is supersaturation of the bile, which in turn gives rise to accumulation and stasis of the bile and the development of gall stones. One of the factors is said to be the hypothyroid state. Hypothyroidism itself is a significantly common endocrine disorder that affects almost every nucleated cell in the body. There is decreased efficacy of the thyroid gland. The serum T3 and T4 levels might be found on the lower side whereas thyroid-stimulating hormone (TSH) values are found to be high. In some of the cases, though the T3 and T4 levels are maintained within the normal limits, the TSH shows raised values, which are labeled as subclinical hypothyroidism. The state of hypothyroidism may act upon the amount of bile secretion, the flow of bile into the intestines, cholesterol metabolism, and the action of the sphincter of Oddi. Studies have shown results pointing towards the correlation between these two factors. The basic mechanism behind the correlation between cholelithiasis and hypothyroidism is supposed to be due to the action of the hypothyroid state on the functioning of the sphincter of Oddi. The hypothyroid state is supposed to be decreasing the tendency of the sphincter of Oddi to relax, thus causing stasis of the bile, which over time leads to initiation of supersaturation of the bile and formation of gall stones. Both subclinical hypothyroidism and clinical hypothyroidism are found to be significantly common in patients having cholelithiasis. We, in this review article, have taken into consideration various studies which have been performed regarding this topic worldwide. The studies have been performed on individuals who are already diagnosed with either of these diseases and are then screened for the presence of the other disease included in this study. The degree of correlation varies according to the location of the stones and their sizes. Though various studies show varying results to some extent, overall almost all the studies show significant pieces of evidence of the correlation between cholelithiasis and hypothyroidism.

## Introduction and background

Cholelithiasis is mentioned as the commonly encountered biliary pathology and gall stones can be pigmented, cholesterol, or mixed. The literature has shown that 2.4% of female patients treated for hypothyroidism had cholecystectomy [1]. The deranged metabolism of cholesterol, the insufficient secretion of bile, and the impaired sphincter of Oddi's mechanism of relaxing the sphincter are considered to contribute to cholelithiasis and common bile duct (CBD) stone in patients having hypothyroidism [2-7]. Volzke et al. studied and put forth the findings on the relation between high thyroid-stimulating hormone (TSH) levels and gall bladder stones disease [8]. In 2003 Laukkarrinen et al. explained the prolonged time of emptying of the biliary tract in cases of hypothyroidism, which is concordant with the findings of Yousif that there is a relationship between cholelithiasis and increased serum TSH levels [7,9]. Up till now, the studies have proven a prevalence of about 8.8% of hypothyroidism (subclinical or clinical) in cholelithiasis patients [7-10]. Gallstones (cholelithiasis) are said to be the most common biliary pathology. Recent study reports have found that 10-15% of the adult population in the United States has gall bladder stones (20 million), 3% of them have undergone cholecystectomy, 85% of them are asymptomatic, 1-4% of them develop symptoms each year, and females are more affected than males in a ratio of 3:1 [11].

The hypothyroidism disease itself can either be of hypothyroidism (overt) or subclinical type, in which the serum level of thyroxine is lesser than the expected normal [12]. In cases of serum thyroid hormones within normal lab level, but serum TSH level slightly raised, subclinical hypothyroidism (mild thyroid failure) is labeled [13]. However, on the other hand, some extra thyroidal effects of it have been reported in the literature [14,15]. The highest incidence rates of cholelithiasis in the world are 21.5/100,000 in females in Delhi [16]. The disease is frequently encountered in otherwise healthy young to middle-aged people with a prevalence being around 11-36 % on the autopsy report. India has a prevalence of hypothyroidism to be around 10.95% [17]. According to a projection from various studies on thyroid disease, about 42 million people in India are estimated to be suffering from one or the other type of thyroid disease [18]. A very important factor in the development of gall stones is the stasis of biliary products, which may be an effect of stenosis of the sphincter of Oddi, dyskinesia, or stricture of the bile duct [19,20]. Both of these modalities, that is the decreased flow of bile and dysfunction of the sphincter of Oddi, are regarded as crucial functional mechanisms that may be promoting the development of cholelithiasis [21]. In studies worldwide, it is found that there is a significant correlation between the two diseases, namely gall bladder stones and hypothyroidism. It has been documented that due to scarcity of the thyroxine hormone, there is decreased relaxation effect (inhibitory effect) on the sphincter of Oddi, which in turn causes stasis of bile and thus leads to the development of gallstones. The presence of gallstones has been found to be approximately 5% to 26% in various countries and areas of the world. The pathogenesis of gallstone disease is not unique but does appear to be multifactorial [22].

The researchers aim to document the correlation between cholelithiasis and hypothyroidism not only in various age groups but also establish a link between subclinical hypothyroidism and cholelithiasis. 

## Review

The process of formation of gall bladder stones is itself a very complex one. There can be numerous factors that can ultimately lead to cholelithiasis in patients having hypothyroidism. The decreased serum thyroxine levels affect the metabolism of cholesterol and that in turn leads to the process of supersaturation. It also affects the filling of the gall bladder, its motility, and contractility of the gall bladder. This in turn leads to the retention of cholesterol in the gall bladder and provokes the nucleation and maturation of the gallbladder stones. The hypothyroid state decreases the secretion of bile, leading to precipitation and formation of the stones. It also decreases the sphincter of Oddi's relaxing tendency, leading to further stasis of the bile as it expresses the thyroid hormone receptors named TR beta 1 and beta 2. A prospective study proved that the subclinical hypothyroidism state also is comparatively more among common bile duct (CBD) stone patients [23]. In 2010, a medical registry-based study from Finland showed that hypothyroid patients did seem to have a higher possibility of the presence of CBD stone treatment [24]. Thyroid hormones have their receptors placed inside the nucleus of the cell; this they need to get transported through the plasma membrane to express their action. It is an important hormone as almost all types of nucleated cells in vertebrates have thyroid hormone receptors and are dependent on them. Recently numerous new transporters have been identified for the thyroid hormone named monocarboxylate transporter MCT10, MCT8, and one anion transporting polypeptide 1C1.

Amount of bile secretion and hypothyroidism

An observation accomplished on a hypothyroid-affected person after thyroidectomy confirmed the equal uptake of the radioactive contrast in the biliary tree depicting that it has no remarkable role in the early stages of hypothyroidism. But research accomplished on rats with the assistance of cannulation of the bile duct displayed a huge impact in the prolonged stages of hypothyroidism; for that reason, it's been taken into consideration that there is supposed to be at least some impact of hypothyroidism in the process of secretion of bile in the long term [12]

Hypothyroidism affects the flow of bile into the intestines

There have been significantly variable findings in radioactive studies which might be because of modifications in the composition of the bile and gall bladder motility and the resistance essentially because of modifications in the sphincter of Oddi contractility. The transport time between the hilum and gall bladder is notably extended in hypothyroid cases. Hypothyroidism decreased and hyperthyroidism elevated the flow rate of the bile into the duodenum [14]

Hypothyroidism and cholesterol metabolism

Most hypothyroid sufferers have raised serum levels of cholesterol and deranged lipid profile which is considered responsive to the treatment of hypothyroidism regardless of concomitant hyperlipidemia. The reduced low-density lipoprotein receptor activity is the risk factor behind the deranged serum levels of cholesterol. Reduced regulation of the hydroxymethylglutaryl-coenzyme A (HMG-CoA) reductase enzyme results in reduced cholesterol synthesis. Hypothyroidism causes decreased cholesterol secretion and the treatment of hypothyroidism leads to accelerated cholesterol secretion. The hypercholesterolemia state leads to supersaturation of cholesterol, in turn inflicting decreased motility, contractility, and atypical filling of the gall bladder. This directly contributes to the nucleation and formation of gallbladder stones [11,12,17]

Hypothyroidism and sphincter of Oddi

The gastrointestinal tract goes into a hypoactive state in a pre-existing hypothyroid state. Thyrogenic hormones have a direct action on the smooth muscles which tends to relax the smooth muscles. Potassium channel blocker glibenclamide attenuates the thyroid hormone effect on smooth muscles and causes further relaxation [20]

Numerous studies [15-22] have been performed to study and establish a correlation between cholelithiasis and hypothyroidism (clinical or subclinical). Studies have shown results showing the correlation between these two pathologies. The studies which were performed on the groups having done cholecystectomy and thyroid function test was done in that patient which showed a significant correlation as there were 11% cases of common bile duct stones which showed hypothyroidism compared to those showing in the control group where there were only 2% of the cases. There were 6% of cases of isolated gall bladder stones excluding the presence of common bile duct stones having a hypothyroidism state. A study confirmed that subclinical hypothyroidism is common among common bile duct stone patients [9]. This study focused on the prevalence of subclinical hypothyroidism, which was previously undiagnosed in clinically euthyroid common bile duct stone patients compared to non-gallstone controls. It was derived that 5.3% of the common bile duct stone patients had subclinical hypothyroidism.

As the process of stone formation in the gall bladder or common bile duct is considerably lengthy, there are very high chances that the process of formation of the stones has already been initiated before even detection of hypothyroidism and initiation of thyroxine supplementations. Thus it has often been stated that both subclinical and clinical hypothyroidism is significantly common in patients having gallbladder or even common bile duct stones. It has also been stated in the literature that findings show significant benefits concerning dyslipidemia, cardiac complications, and neuromuscular symptoms if the patients are treated with thyroxine supplementation, especially in subclinical cases. Despite all these results from various studies, there are still questions about the mechanism behind the association of hypothyroidism and cholelithiasis though a significant correlation has been proved. The thyroid hormone has numerous effects on metabolism and also on cholesterol metabolism, which is frequently observed with the presence of deranged values of lipid profile in this kind of patient. In cases of hypothyroidism when the metabolism of cholesterol has been affected, the level of serum cholesterol rises leading to supersaturation in the bile [25,26]. The slowed metabolism and functions of the body may also lead to decreased motility of the gallbladder, depressed intensity of contractility, impaired contractility of the sphincter of Oddi, and abnormal filling [25-27]. This leads to a longer duration of stasis of bile in the gall bladder. This leads to the accumulation of cholesterol crystals, with enough time for nucleation and maturation of gallstones [28]. With this, the bile secretion rate is decreased, impairing the clearing of the gallbladder and bile ducts.

The serum thyroxine-secreting hormone values in cholelithiasis patients have been studied. The patients with gallbladder and CBD stones will have an equal increase in the prevalence of subclinical hypothyroidism if the absence of T4 affects the cholesterol metabolism and hepatic biliary secretion. In some of the studies, it was observed that CBD stone patients have twice the chance of being diagnosed with hypothyroid as compared to patients with gallbladder stones [28,29]. This can be due to the decreased pro-relaxing effect of the T4 hormone resulting in slower emptying of the biliary duct into the duodenum, which may give rise to CBD stones [26,28]. One of the most important risk factors for gallstones is age. It appears that the cut-off between relatively high and low rates of cholecystectomies can be conveniently denoted by the age of 40 years. The incidence increases to about four times between the ages of 40 and 69 years. Laukkarinen et al. study denotes that mild and preclinical thyroid function abnormalities also should be screened in patients diagnosed with gallstones, especially in women who have crossed 60 years of age [4]. With increasing age, the decrease in the water contents of the body, which may reach 45% of body weight, due to a decrease in lean (muscle) mass of the body, may lead to concentration of body fluids and excretions and more deposition and accumulation of solid contents of the excretions, which lead to nucleation and maturation of gall stone [29]. Völzke et al. in their study proved the correlation between the presence of raised TSH levels and sonographically detected gallstones [30]. A total of 14.4% of patients were found to have cholelithiasis in a study conducted by Kotwal and Rinchen in Sikkim, India, on hypothyroidism patients. There was statistical significance as in patients having hypothyroidism, cholesterol stones were found with higher prevalence [29]. Similar findings were corroborated in Ibrahim and Abdulbary's study and the study by Yousif [30,9]. Table 1 shows the comparison between various studies performed in the past [23,4,7].

**Table 1:** Data of the studies published in past years, reporting an association between hypothyroidism and gall bladder/CBD stones

Author	Year	Journal	Study Types	Patients Population	Patient Number	Gender	Age	Finding	Risk Factor
Inkinen et al. [23]	2001	Hepatogastroenterol	Retrospective study	Age and sex-matched cholelithiasis patients, CBD stone patients and controls	168	65%F	>60	Hypothyroidism: CBD stones 11% cases, GB stones 6% cases, controls 2%	Groups were matched
Laukkarinen et al. [4]	2007	J Clin Endocrinol Metab	Multicentric, prospective study	Euthyroid CBD stone cases and non-gallstone controls	445	61%F	Median 67	Subclinical hypothyroidism cases (TSH > 6.0 mU/L): CBD stones cases 5.3%, control 1	Groups were matched
Laukkarinen et al. [7]	2010	Scand J Gastroenterol	Cohort study, based on medical registry	Patients diagnosed with hypothyroidism and sex, age, and residence adjusted glaucoma controls	28.668	68%F	Median 62	After diagnosing hypothyroidism 56% more CBD stone treatments than after diagnosing glaucoma	Patients with isolated hypothyroidism (or glaucoma) cohort of patients. Also no difference in any other medications between the groups.

In a randomized prospective study, Mulita et al. also found that a total of 18 out of 316 (5.7%) patients who underwent laparoscopic cholecystectomy because of cholelithiasis had hypothyroidism [31].

## Conclusions

In summary, research studies suggest that there's a significant association between clinical or subclinical hypothyroidism state and the development of common bile duct stones. Particularly the modifications in the functioning of the sphincter of Oddi underline the association between cholelithiasis and hypothyroidism. Though the prevalence of the stones in the common bile duct in cases of hypothyroidism is more as compared to the ones having isolated gall bladder stones, the correlation of hypothyroidism with gall bladder stones is still significant and needs to be focused upon. There is evidence pointing in the direction of the phenomenon of reduction of bile flow because of the absence or scarcity of thyroid hormone. The thyroid hormone acts on the intranuclear receptors and this has an effect on almost all the nucleated cells in the human body, showing widespread effects. The state of raised thyroid stimulating hormone causes numerous effects, ultimately leading to the precipitation of gallstone disease. There are many studies enlightening the need for screening hypothyroid patients for possible gallbladder stones. Apart from the increased cholesterol concentration and reduced bile flow rate, the pro-relaxant action of thyroid hormones at the sphincter of Oddi plays a critical function in the improvement of the disease. The stone formation in particular initiates during the early untreated phase of the hypothyroid state, which continues to the level of maturation even after the initiation of treatment for hypothyroidism. Cardiovascular, neuromuscular, and dyslipidemia pathologies can be prevented through thyroxine supplementations. Most importantly, at the same time as treating sufferers with common bile duct stones or microlithiasis, clinicians need to preserve in thought the possible synchronous existence of hypothyroidism and for that reason, the thyroid workup needs to be done as a routine practice.
